# Density Functional Theory Study of Triple Transition Metal Cluster Anchored on the C_2_N Monolayer for Nitrogen Reduction Reactions

**DOI:** 10.3390/molecules29143314

**Published:** 2024-07-13

**Authors:** Shifa Xiao, Daoqing Zhang, Guangzhao Wang, Tianhang Zhou, Ning Wang

**Affiliations:** 1 College of Physics Science and Technology, Lingnan Normal University, Zhanjiang 524048, China; 2 Key Laboratory of Extraordinary Bond Engineering and Advanced Materials Technology of Chongqing, School of Electronic Information Engineering, Yangtze Normal University, Chongqing 408100, China; 3 College of Carbon Neutrality Future Technology, China University of Petroleum (Beijing), Beijing 102249, China; 4 State Key Laboratory of Heavy Oil Processing, China University of Petroleum (Beijing), Beijing 102249, China; 5 School of Science, Key Laboratory of High Performance Scientific Computation, Xihua University, Chengdu 610039, China

**Keywords:** nitrogen reduction reaction, density functional theory, C_2_N, triple transition metal, catalytic activity, catalytic selectivity

## Abstract

The electrochemical nitrogen reduction reaction (NRR) is an attractive pathway for producing ammonia under ambient conditions. The development of efficient catalysts for nitrogen fixation in electrochemical NRRs has become increasingly important, but it remains challenging due to the need to address the issues of activity and selectivity. Herein, using density functional theory (DFT), we explore ten kinds of triple transition metal atoms (M_3_ = Sc, Ti, V, Cr, Mn, Fe, Co, Ni, Cu, and Zn) anchored on the C_2_N monolayer (M_3_-C_2_N) as NRR electrocatalysts. The negative binding energies of M_3_ clusters on C_2_N mean that the triple transition metal clusters can be stably anchored on the N6 cavity of the C_2_N structure. As the first step of the NRR, the adsorption configurations of N_2_ show that the N_2_ on M_3_-C_2_N catalysts can be stably adsorbed in a side-on mode, except for Zn_3_-C_2_N. Moreover, the extended N-N bond length and electronic structure indicate that the N_2_ molecule has been fully activated on the M_3_-C_2_N surface. The results of limiting potential screen out the four M_3_-C_2_N catalysts (Co_3_-C_2_N, Cr_3_-C_2_N, Fe_3_-C_2_N, and Ni_3_-C_2_N) that have a superior electrochemical NRR performance, and the corresponding values are −0.61 V, −0.67 V, −0.63 V, and −0.66 V, respectively, which are smaller than those on Ru(0001). In addition, the detailed NRR mechanism studied shows that the alternating and enzymatic mechanisms of association pathways on Co_3_-C_2_N, Cr_3_-C_2_N, Fe_3_-C_2_N, and Ni_3_-C_2_N are more energetically favorable. In the end, the catalytic selectivity for NRR on M_3_-C_2_N is investigated through the performance of a hydrogen evolution reaction (HER) on them. We find that Co_3_-C_2_N, Cr_3_-C_2_N, Fe_3_-C_2_N, and Ni_3_-C_2_N catalysts possess a high catalytic activity for NRR and exhibit a strong capability of suppressing the competitive HER. Our findings provide a new strategy for designing NRR catalysts with high catalytic activity and selectivity.

## 1. Introduction

Ammonia (NH_3_) is an important raw chemical material that plays an essential role in industry, agricultural production, energy storage and conversion, and other fields [1,2,3]. At present, industrial ammonia synthesis mainly relies on the traditional Haber–Bosch process [4,5]. Since this technology requires high temperatures and pressure, it not only consumes vast energy, but also emits a large amount of greenhouse gasses. Therefore, against the backdrop of the energy crisis and increasing environmental concerns, developing new processes for efficiently synthesizing ammonia under mild conditions is urgent [6].

Compared with the Haber–Bosch method, the electrocatalytic approach for achieving nitrogen reduction reactions (NRRs) can theoretically be carried out at room temperature and pressure [7,8]. Meanwhile, the sources of raw water and nitrogen are extensive, which provides an opportunity for achieving the green synthesis of ammonia under mild conditions [9]. In recent years, electrocatalytic nitrogen reduction for ammonia production has attracted significant attention, and related research has shown a rapid growth trend [10,11,12,13]. However, the current study shows that although electrocatalytic technology can achieve the green synthesis of ammonia, the thermodynamic and kinetic obstacles to the production of ammonia via electrocatalytic nitrogen reduction at room temperature and pressure are enormous due to the high stability of the N≡N triple bond and the slow adsorption of nitrogen [14,15]. Moreover, the selectivity of the nitrogen reduction reaction and the ammonia production rate are greatly reduced due to the hydrogen precipitation competition reaction [16]. Therefore, how to improve the ammonia production rate and the catalyst selectivity at the same time is the biggest challenge in the study of electrocatalytic nitrogen reduction at ambient temperature and pressure.

The two-dimensional material known as C_2_N has recently emerged as a subject of interest among researchers, thanks to its remarkable stability, cavity structure, high specific surface area, and other distinctive attributes [17,18,19,20]. Its spacious cavities make C_2_N a suitable support for anchoring metal atoms to catalyze various chemical reactions [21]. Metal clusters featuring exposed atomic interfaces and distinct electronic configurations have garnered significant attention in multiphase catalysis [22]. Transition metals like Fe-, Ru-, and Co-based complexes, with their d-orbitals capable of donating electrons to the empty π*-orbitals of N_2_ and accepting electrons from its σ-orbitals, enhance N_2_ adsorption, making them suitable for nitrogen reduction reaction (NRR) catalysis [23,24,25,26]. In response to the rising interest in single-atom catalysts for efficient NRR electrocatalysts [27,28,29], dual-atom and triple-atom catalysts have also been experimentally and theoretically explored for their catalytic performance in NRRs [30,31,32]. For instance, the work proposed by Chen et al. indicates that due to the unique characteristics of M_3_ (M = Mn, Fe, Co, and Ni) active sites, the triple-atom catalysts exhibit better catalytic activity towards NRRs than single-atom and double-atom catalysts [33]. Liu et al. studied Fe_3_ clusters anchored on the surface of Al_2_O_3_ as multiphase catalysts for NRRs [34]. They discovered that their comparable activity to Ru catalysts, which is attributed to the large spin polarization, low iron oxidation state, and multi-step oxidation–reduction ability of Fe_3_ clusters.

Motivated by the above studies, a series of triple transition metal atoms (M_3_ = 3d transition metal, Sc, Ti, V, Cr, Mn, Fe, Co, Ni, Cu, and Zn) anchored on the C_2_N monolayer (M_3_-C_2_N) are designed as electrocatalysts for NRRs, and the electronic structures and NRR catalytic mechanisms are systematically investigated using density functional theory (DFT). Firstly, the binding energy of triple transition metal atoms on C_2_N is calculated to evaluate the stability of M_3_-C_2_N catalysts to prescreen the promising candidates for NRR catalysts. The negative binding energies manifest that the triple transition metal clusters can be stably anchored on the N6 cavity of the C_2_N monolayer. Next, as the first step of the NRR, the adsorption and activation of N_2_ molecules on the surface of M_3_-C_2_N are studied via the adsorption structure, adsorption energy, and electronic structures. Moreover, the catalytic activity and mechanisms are systematically investigated based on the Gibbs free energy of the whole NRR process. According to the limiting potential, we screen out four highly active NRR catalysts, including Co_3_-C_2_N, Cr_3_-C_2_N, Fe_3_-C_2_N, and Ni_3_-C_2_N, while they can suppress the competitive hydrogen evolution reaction. Among them, Co_3_-C_2_N exhibits the highest NRR activity with a limiting potential of −0.61 V. This study provides a comprehensive understanding of the stability, activity, and selectivity of M_3_-C_2_N as NRR catalysts, which can guide the further experimental exploration of M_3_-C_2_N or other related reactions.

## 2. Results and Discussion

### 2.1. Structure and Stability of M_3_-C_2_N

**Figure 1:**
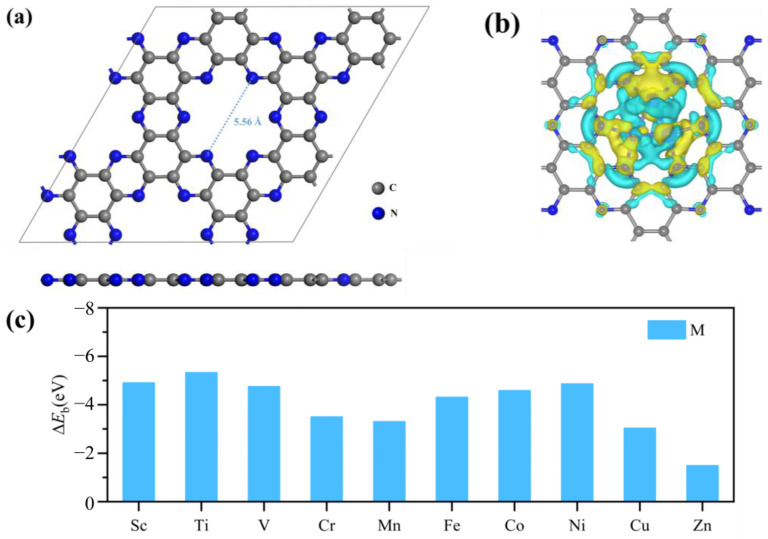
(**a**) Optimized structure of C_2_N. (**b**) The charge density difference of Co_3_-C_2_N. (Isosurface = 0.003 e/Bohr^3^. Yellow represents the charge increase, and cyan represents the charge decrease.) (**c**) The binding energy of triple transition metal atoms on the C_2_N monolayer.

The pristine C_2_N monolayer containing 48 C and 24 N atoms is optimized, and the optimized lattice constant is *a* × *b* = 16.83 Å × 16.77 Å, which is consistent with the previous literature [35,36]. As shown in Figure 1a, the N6 cavity of the C_2_N structure is 5.56 Å, which is large enough to anchor triple metal atoms. Moreover, the N atoms surrounding the N6 cavity exhibit electron-rich properties, and due to the electron loss of the C atom, the N atom is negatively charged. Therefore, N atoms endow the N6 cavity environment with high electron enrichment properties, making the cavity an ideal place to contain positively charged TM ions. Figure 1b exhibits the optimized structure of Co triple metal clusters anchored on the N6 cavity of C_2_N, and other optimized structures of M_3_-C_2_N are displayed in the Supplementary Materials (Figure S1). The average bond length of the Co-N bond is 1.89 Å, indicating a strong interaction between Cu and N atoms. In addition, the difference in charge density clearly shows the accumulation and depletion of charges around the Co and adjacent N atoms. It is worth noting that the high charge density around the Co atoms facilitates the subsequent adsorption of N_2_ molecules. To evaluate the stability of the M_3_-C_2_N catalysts, the binding energy of triple transition metal atoms on the C_2_N monolayer is calculated, as displayed in Figure 1c. The ∆*E*_b_ values of all catalysts are less than 0 eV, confirming the stability of the M_3_-C_2_N system and the triple transition metal clusters stably anchored on the N6 cavity of the C_2_N monolayer.

### 2.2. N_2_ Adsorption on M_3_-C_2_N

**Figure 2:**
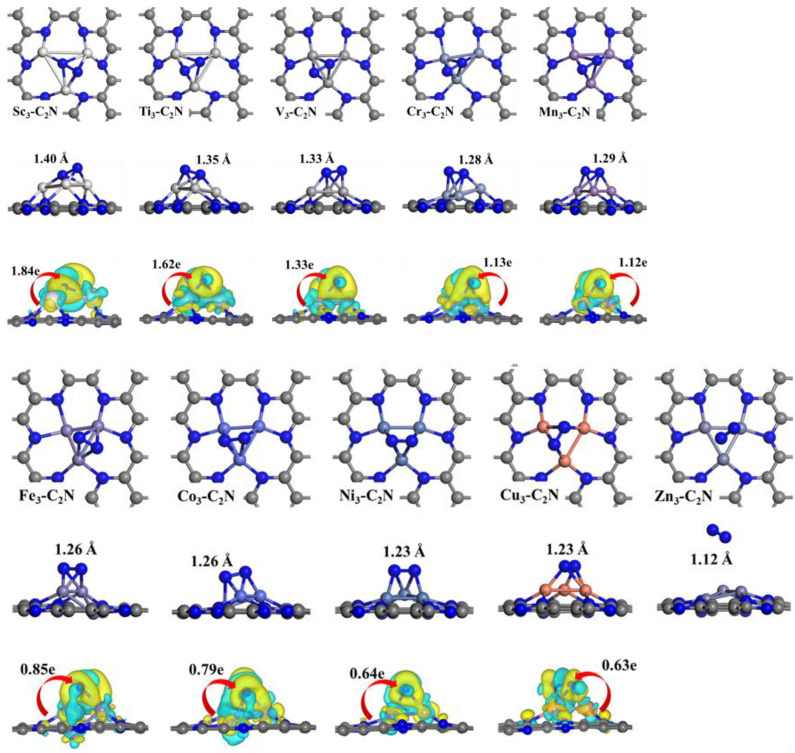
Optimized adsorption configurations and charge density differences of N_2_ adsorbed on M_3_-C_2_N. The isosurface value is 0.003 e/Bohr^3^.

**Figure 3:**
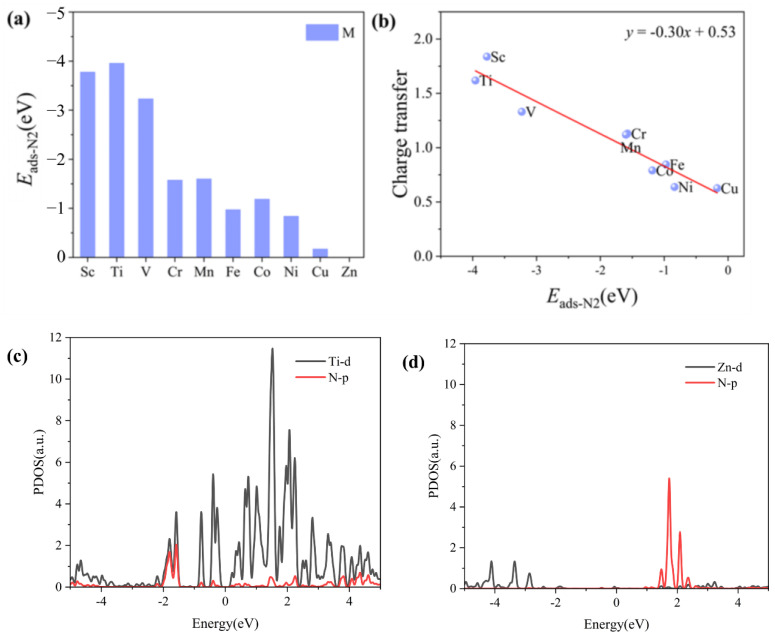
(**a**) Adsorption energies of N_2_ on the M_3_-C_2_N catalysts. (**b**) Charge transfer from M_3_-C_2_N to the adsorbed N_2_ as a function of *E*_ads-N2_. The partial density of states (PDOS) of N_2_ on (**c**) Ti_3_-C_2_N and (**d**) Zn_3_-C_2_N.

For the whole NRR process, the adsorption and activation of N_2_ molecules on the catalyst surface is a crucial step. Therefore, the adsorption performance of N_2_ on the surface of M_3_-C_2_N catalysts is investigated, and the adsorption configurations are shown in Figure 2. It can be seen that except for on Zn_3_-C_2_N, N_2_ is more energetically favorable when adsorbed in the side-on mode on all M_3_-C_2_N, which is consistent with the research findings in the literature. Compared with the N-N bond length (1.12 Å) of free N_2_ molecule, the N-N bond length of N_2_ after adsorption has been elongated to varying degrees, indicating that N_2_ has been activated on M_3_-C_2_N. On Zn_3_-C_2_N, the bond length of N-N is still 1.12 Å, and N_2_ is far from the catalyst surface, indicating that the N_2_ molecule cannot be adsorbed on it, so the NRR activity of Zn_3_-C_2_N will not be discussed later. In addition, the charge density differences plot also suggests that the adsorbed N_2_ interacts with M_3_-C_2_N, activating the N≡N triple bond. The adsorption energies of N_2_ on the surface of M_3_-C_2_N catalysts are summarized in Figure 3a. It can be seen that the adsorption of N_2_ on Sc_3_-C_2_N, Ti_3_-C_2_N, and V_3_-C_2_N is exceedingly strong, and the *E*_ads-N2_ values are −3.78 eV, −3.96 eV, and −3.23 eV, respectively. The adsorption strength of N_2_ on Cu_3_-C_2_N is the most weak with only −0.17 eV values of *E*_ads-N2_. The range of adsorption energy values on the other six catalysts is from −0.84 eV to −1.60 eV. To further elucidate the adsorption and activation of N_2_, the scaling relationship between the charge transfer from M_3_-C_2_N to the adsorbed N_2_ and the adsorption energy *E*_ads-N2_ is studied and displayed in Figure 3b. It can be seen that the most charge transfer is from M_3_-C_2_N to N_2_ on Sc_3_-C_2_N, Ti_3_-C_2_N, and V_3_-C_2_N, corresponding to 1.84e, 1.62e, and 1.33e, respectively, which is consistent with the adsorption strength of them and the N-N bond length of adsorbed N_2_. There is a significant positive correlation between charge transfer and *E*_ads-N2_. That is to say, the more charge transfer, the stronger the N_2_ adsorption, and the more negative the value of *E*_ads-N2_. In order to better understand the adsorption of N_2_, the project density of states of the M_3_-C_2_N after adsorption of N_2_ is shown in Figure 3c,d, taking the strongest adsorption on Ti_3_-C_2_N and the weakest adsorption on Zn_3_-C_2_N as examples. It can be seen that for Ti_3_-C_2_N, there is a significant orbital hybridization between the Ti-d and N-p orbitals. For Zn_3_-C_2_N, due to weak adsorption, the N_2_ molecule still maintains a high DOS without orbital hybridization with Zn-d.

### 2.3. NRR Mechanism and Activity on M_3_-C_2_N

**Figure 4:**
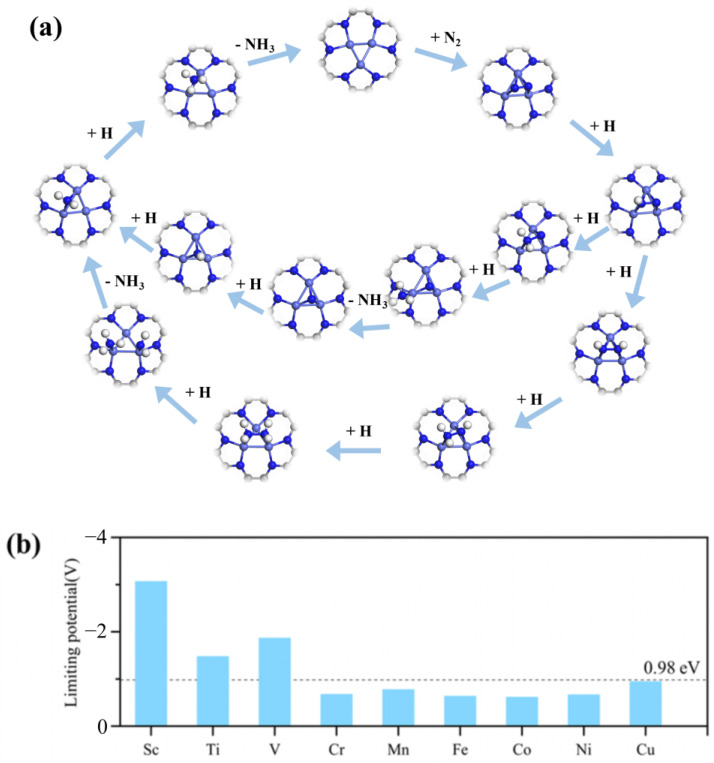
(**a**) Schematic diagram of the NRR catalyzed by Co_3_-C_2_N and the corresponding adsorption intermediate. (**b**) Theoretical limiting potential U_L_ of M_3_-C_2_N.

**Figure 5:**
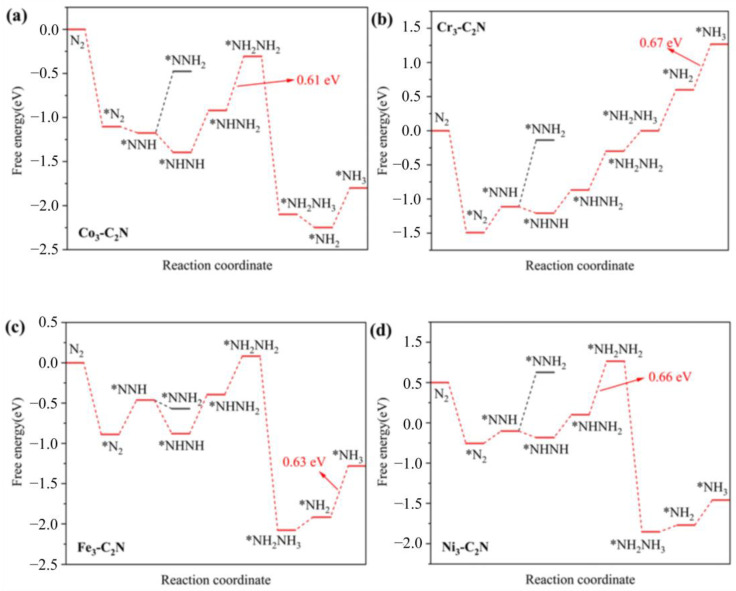
Gibbs free energy diagrams for NRR on (**a**) Co_3_-C_2_N, (**b**) Cr_3_-C_2_N, (**c**) Fe_3_-C_2_N, and (**d**) Ni_3_-C_2_N, respectively.

The dissociation and association pathways are the two common mechanisms for electrocatalytic NRRs. For the dissociation pathway, it is difficult for the catalyst to break the N≡N bond of the adsorbed N_2_ molecule. Therefore, only the association pathway on M_3_-C_2_N is investigated in this work. For the association pathway, before the formation of the first NH_3_ molecule, the two N atoms of N_2_ remain bound to each other. It can be further divided into the distal and consecutive mechanisms (the protons continuously attack a N atom until the first NH_3_ molecule is produced), as well as the alternating and enzymatic mechanisms (the protons alternately bind the two N atoms). Herein, the two NRR mechanisms on Co_3_-C_2_N are shown in Figure 4a, and the adsorbed N_2_ molecule is gradually hydrogenated to produce NH_3_ gas. As we all know, the activity of electrocatalysts can be estimated by the limit potential (U_L_). Therefore, the U_L_ values of the M_3_-C_2_N catalysts are calculated and displayed in Figure 4b, and the Ru(0001) (U_L_ = 0.98 eV) catalyst is chosen as a benchmark to evaluate the electrocatalytic NRR activity of M_3_-C_2_N due to it having the highest theoretical activity on the surface of the bulk metal. It can be seen that the U_L_ values of Sc_3_-C_2_N, Ti_3_-C_2_N, and V_3_-C_2_N are larger than that of Ru(0001), indicating their poor catalytic activity for NRR. The other six M_3_-C_2_N catalysts with U_L_ values less than 0.98 eV exhibit a better catalytic activity than Ru(0001). It is worth noting that the limit potential of Co_3_-C_2_N, Cr_3_-C_2_N, Fe_3_-C_2_N, and Ni_3_-C_2_N are relatively small, and the corresponding values are −0.61 V, −0.67 V, −0.63 V, and −0.66 V, so their free energy diagrams for NRRs are detailed in Figure 5. It can be seen that for Co_3_-C_2_N, Cr_3_-C_2_N, Fe_3_-C_2_N, and Ni_3_-C_2_N, the step of *NNH → *NHNH is more significant downhill than the *NNH → *NNH_2_ step, indicating that the alternating and enzymatic mechanisms on them are more energetically advantageous. In addition, the first step of the NRR shows goes downhill for all four M_3_-C_2_N catalysts, precisely because of the strong N_2_ adsorption, which also suggests that the adsorption and activation of N_2_ molecules can occur at room temperature. The potential-limiting step (PDS) on Co_3_-C_2_N and Ni_3_-C_2_N is the *NHNH_2_ → *NH_2_NH_2_ step, and the corresponding Δ*G* values are 0.61 eV and 0.66 eV. For Cr_3_-C_2_N and Fe_3_-C_2_N, the final step of hydrogenation *NH_2_ → *NH_3_ is the PDS; and their Δ*G* values are 0.67 eV and 0.63 eV. Therefore, Co_3_-C_2_N, Cr_3_-C_2_N, Fe_3_-C_2_N, and Ni_3_-C_2_N are the candidates for NRR catalysts, and Co_3_-C_2_N possesses the highest NRR activity.

### 2.4. Selectivity Evaluation for NRR on M_3_-C_2_N

**Figure 6:**
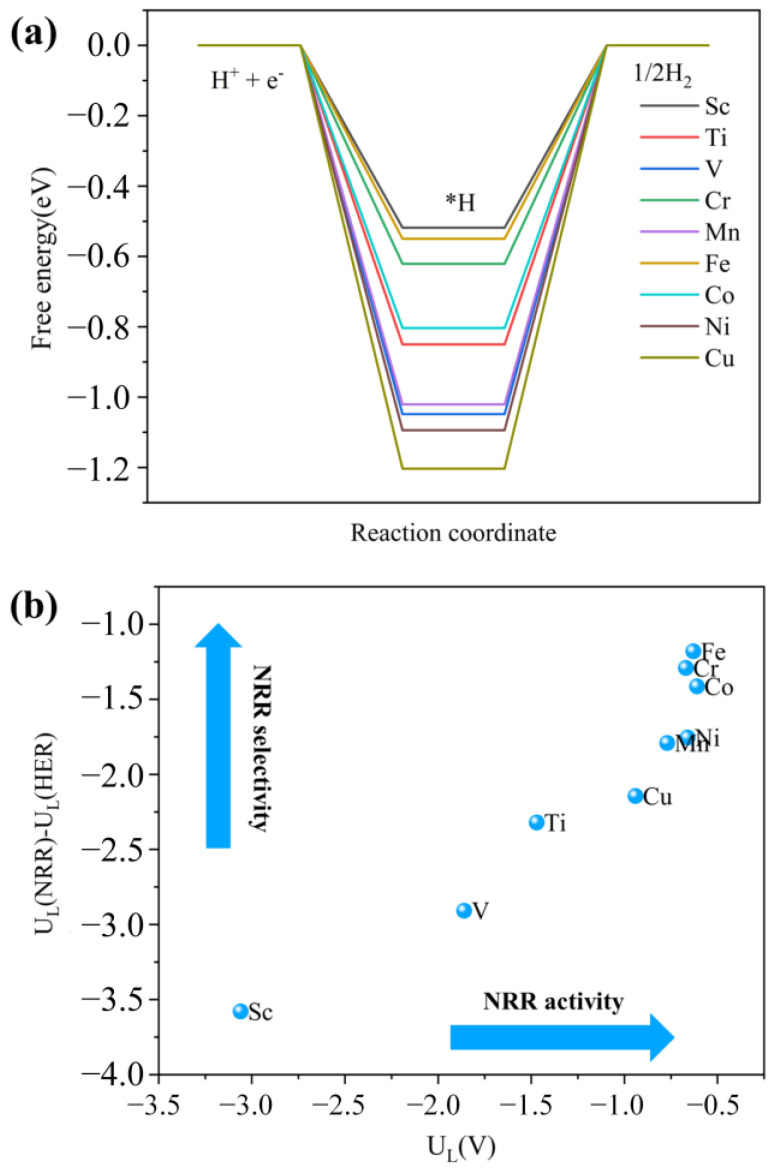
(**a**) Free energy diagrams for the HER on M_3_-C_2_N. (**b**) Liming potential (U_L_) versus U_L_(NRR)—U_L_(HER) on M_3_-C_2_N.

Furthermore, an ideal electrocatalyst for NRR should possess a high stability and activity and effectively suppress the hydrogen evolution reaction (HER) to achieve high production for NH_3_. The HER is the most problematic yet dominant side reaction in the NRR. Therefore, the adsorption free energy of H (ΔG_*H_) on M_3_-C_2_N catalysts is calculated and summarized in Figure 6a. If the ΔG_*H_ values are close to 0 eV, it means that H* cannot easily cover the metal surface and will not block the active sites for NRRs. Although, the ΔG_*H_ values on all M_3_-C_2_N are lower than those on Ru(0001) (ΔG_*H_ = −0.35 eV), some M_3_-C_2_N exhibit a relatively high HER activity, indicating that the HER process that occurred on some M_3_-C_2_N surfaces could be hindered effectively. In addition, the difference in limiting potential between the NRR and HER (U_L_(NRR)—U_L_(HER)) is calculated to estimate the catalytic selectivity for NRR on M_3_-C_2_N; the scaling relationship between the U_L_(NRR)—U_L_(HER) and U_L_(NRR) is plotted in Figure 6b. Notably, Mn_3_-C_2_N, Co_3_-C_2_N, Cr_3_-C_2_N, Fe_3_-C_2_N, and Ni_3_-C_2_N have a relatively high NRR selectivity. Therefore, Co_3_-C_2_N, Cr_3_-C_2_N, Fe_3_-C_2_N, and Ni_3_-C_2_N not only possess a high NRR activity, but also exhibit the highest selectivity for NRRs.

## 3. Computational Methods

All computational studies were executed utilizing the Perdew−Burke−Ernzerhof (PBE) functional [37] implemented in the Vienna Ab Initio Simulation Package (VASP 5.4.4) [38,39]. Spin polarization was incorporated in all calculations, and the electron–ion interactions were described through the projector-augmented wave method with a 450 eV cutoff energy. Atomic structures were fully relaxed until the force on each atom was smaller than 0.02 eV/Å, while the energy convergence was set to 10^−5^ eV. To account for van der Waals (vdW) interactions, Grimme’s DFT-D3 approach was implemented [40]. For geometry relaxation, a 3 × 3 × 1 k-point grid centered at the gamma point was employed, and a 20 Å vacuum space along the z-direction was introduced to prevent periodic image interactions. To simulate the electrolyte solution and address solvation effects, the VASPsol code with an implicit solvation model was utilized [41,42].

The binding energy of triple transition metal atoms on a C_2_N monolayer is calculated to evaluate the stability of M_3_ on the C_2_N monolayer:

∆*E*_b_ = (*E*_M3-C2N_ − *E*_C2N_ − 3*E*_M_)/3 (1)

where *E*_M3-C2N_, *E*_C2N_, and *E*_M_ are the total energies of M_3_-C_2_N, C_2_N, and metal atoms, respectively.

The adsorption energies of reaction species on the M_3_-C_2_N catalyst are determined by:

*E*_ads_ = *E*_tot_ − *E*_species_ − *E*_M3-C2N_ (2)

where *E*_tot_ and *E*_species_ are the total energies of the M_3_-C_2_N with adsorbed reaction species and the isolated reaction species.

Based on the computational hydrogen electrode (CHE) model given by Nørskov and coworkers, the Gibbs free energy change (∆G) for each fundamental step of the NRR is obtained by using the following equation:

Δ*G* = Δ*E* + ΔZPE − *T*Δ*S* (3)

where Δ*E* is the reaction energy difference in each hydrogenation step in the NRR pathways. ΔZPE and *T*Δ*S* are the changes in zero-point energy and entropy (T = 298.15 K), respectively.

Limiting potential (U_L_) was obtained from the maximum free energy change (Δ*G*_max_) among all elementary steps along the lowest-energy pathway by:

U_L_ = −Δ*G*_max_/e (4)

## 4. Conclusions

In summary, the triple transition metal atoms (M_3_ = Sc, Ti, V, Cr, Mn, Fe, Co, Ni, Cu, and Zn) anchored on the C_2_N monolayer (M_3_-C_2_N) as electrocatalysts for NRRs are systematically investigated. Based on the negative binding energies of triple transition metal clusters on C_2_N, the conclusion can be drawn that these ten metal clusters can be stably anchored on the N6 cavity of the C_2_N structure. The N_2_ adsorption results indicate that except for Zn_3_-C_2_N, the N_2_ molecule can be stably adsorbed in the side-on mode on all the M_3_-C_2_N catalysts. In addition, the results combining adsorption configurations and electronic structure demonstrate that the charges transfer from the M_3_-C_2_N to N_2_, activating the N_2_ molecule. The positive correlation of the scaling relationship between the charge transfer and the adsorption energy illustrates that the more charge transfer, the stronger the N_2_ adsorption. The analysis of Gibbs free energy changes suggests that the alternating and enzymatic mechanisms of the association pathway on Co_3_-C_2_N, Cr_3_-C_2_N, Fe_3_-C_2_N, and Ni_3_-C_2_N, which have the relatively low limiting potentials of −0.61 V, −0.67 V, −0.63 V, and −0.66 V, are more energetically advantageous. Moreover, the potential-limiting step (PDS) on both Co_3_-C_2_N and Ni_3_-C_2_N is the *NHNH_2_ → *NH_2_NH_2_ step, while that on Cr_3_-C_2_N and Fe_3_-C_2_N is the step of *NH_2_ → *NH_3_. Finally, we investigate the competitive reaction of HER on M_3_-C_2_N, and it can be concluded that five catalysts (including Mn_3_-C_2_N, Co_3_-C_2_N, Cr_3_-C_2_N, Fe_3_-C_2_N, and Ni_3_-C_2_N) have a relatively high NRR selectivity. Overall, Co_3_-C_2_N, Cr_3_-C_2_N, Fe_3_-C_2_N, and Ni_3_-C_2_N catalysts can effectively inhibit the competitive HER with a favorable limiting potential. We hope this work can provide a valuable clue for the experimental explorations of the triple transition metal clusters anchored on C_2_N catalysts and can promote more experimental and theoretical methods to design new high-performance NRR catalysts for efficient NH_3_ production.

## Data Availability

The raw data supporting the conclusions of this article will be made available by the authors on request.

## Supplementary Materials

The following supporting information can be downloaded at: https://www.mdpi.com/article/10.3390/molecules29143314/s1, Figure S1. Optimized structures of M_3_-C_2_N.
