# Supplementary material for: Density Functional Theory Study of Triple Transition Metal Cluster Anchored on the C2N Monolayer for Nitrogen Reduction Reactions
Source: Molecules. 2024 Jul 13;29(14):3314. doi: 10.3390/molecules29143314 (PMC11280456; doi:10.3390/molecules29143314)
Supplement: Supplementary file 1 [file molecules-29-03314-s001.zip › molecules-3100945-supplementary.pdf]

Supplementary Material

Density functional theory study of triple transition metal cluster anchored on the C_2N monolayer for nitrogen reduction reaction

Shifa Xiao [1], Daoqing Zhang [1], Guangzhao Wang [2], Tianhang Zhou [3,4,*] and Ning Wang [5,*]

[1] College of Physics Science and Technology, Lingnan Normal University, Zhanjiang 524048, China; xiaosf@lingnan.edu.cn (S.X.); zhangdq@lingnan.edu.cn (D.Z.)
[2] Key Laboratory of Extraordinary Bond Engineering and Advanced Materials Technology of Chongqing, School of Electronic Information Engineering, Yangtze Normal University, Chongqing 408100, China; wangyan6930@126.com
[3] College of Carbon Neutrality Future Technology, China University of Petroleum (Beijing), Beijing 102249, China
[4] State Key Laboratory of Heavy Oil Processing, China University of Petroleum (Beijing), Beijing 102249, China
[5] School of Science, Key Laboratory of High Performance Scientific Computation, Xihua University, Chengdu 610039, China
* Correspondence: zhouth@cup.edu.cn (T.Z.); ningwang0213@163.com (N.W.)

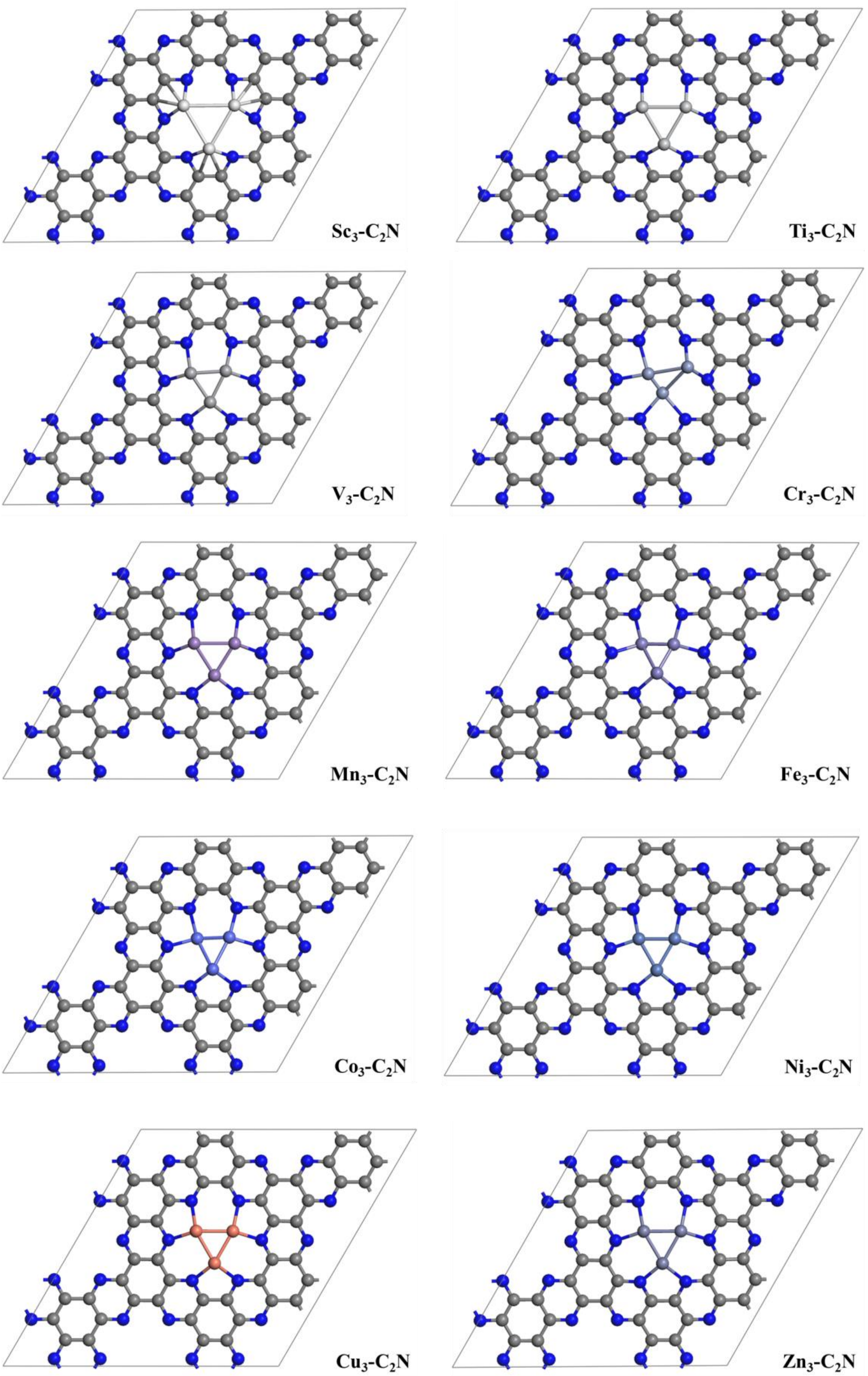

Fig. S1. Optimized structures of M_3-C_2N.
